# Identification of the Volatile Compounds and Observation of the Glandular Trichomes in *Opisthopappus taihangensis* and Four Species of *Chrysanthemum*

**DOI:** 10.3390/plants9070855

**Published:** 2020-07-06

**Authors:** Yanhong Guo, Tengxun Zhang, Jian Zhong, Tingting Ba, Ting Xu, Qixiang Zhang, Ming Sun

**Affiliations:** 1 Beijing Key Laboratory of Ornamental Plants Germplasm Innovation and Molecular Breeding, National Engineering Research Center for Floriculture, Beijing Laboratory of Urban and Rural Ecological Environment, Key Laboratory of Genetics and Breeding in Forest Trees and Ornamental Plants of Ministry of Education, School of Landscape Architecture, Beijing Forestry University, Beijing 100083, China; guoyanhong@bjfu.edu.cn (Y.G.); zhangtx2017@bjfu.edu.cn (T.Z.); zhongjian@bjfu.edu.cn (J.Z.); batingting2020@bjfu.edu.cn (T.B.); tingx_2020@bjfu.edu.cn (T.X.); zqx@bjfu.edu.cn (Q.Z.); 2 Beijing Advanced Innovation Center for Tree Breeding by Molecular Design, Beijing Forestry University, Beijing 100083, China

**Keywords:** volatile compounds, GC-MS, trichomes, density, histochemical, correlation

## Abstract

*Opisthopappus taihangensis* (Ling) Shih, a wild relative germplasm of chrysanthemum, releases a completely different fragrance from chrysanthemum species. We aimed to identify the volatile compounds of the leaves of *O. taihangensis* and four other *Chrysanthemum* species using headspace solid-phase micro-extraction combined with gas chromatography-mass spectrometry (HS-SPME-GC/MS). In total, 70 compounds were detected, and terpenoids accounted for the largest percentage in these five species. Many specific compounds were only emitted from *O. taihangensis* and not from the other four species. In particular, 1,8-cineole could be responsible for the special leaf fragrance of *O. taihangensis* as it accounted for the largest proportion of the compounds in *O. taihangensis* but a small or no proportion at all in other species. The glandular trichomes (GTs) in the leaves are the main organs responsible for the emission of volatiles. To explore the relationship between the emissions and the density of the GTs on the leaf epidermis, the shape and density of the GTs were observed and calculated, respectively. The results showed that the trichomes have two shapes in these leaves: T-shaped non-glandular trichomes and capitate trichomes. Histochemical staining analyses indicated that terpenoids are mainly emitted from capitate glandular trichomes. Correlation analysis showed that the volatile amount of terpenoids is highly related to the density of capitate trichomes. In *O. taihangensis*, the terpenoids content and density of capitate trichomes are the highest. We identified the diversity of leaf volatiles from *O. taihangensis* and four other *Chrysanthemum* species and found a possible relationship between the content of volatile compounds and the density of capitate trichomes, which explained the cause of the fragrance of *O. taihangensis* leaves.

## 1. Introduction

The aroma of plants is produced by a series of complex low-molecular-weight substances (typically less than 300 Da), which are a class of lipophilic substances with an aromatic odor [1]. The release of aroma is not only an important expression signal of the pollination and fertilization of plants, but also an adaptation mechanism of plants that allows them to actively defend against herbivores [2]. In recent years with the continuous development of aromatic volatile compound extraction and detection technology, more than 2000 kinds of volatile organic compounds (VOCs) from 90 families, belonging to 991 species and subspecific taxa, have been identified [3]. These VOCs are classified into terpenoids, phenylpropanoids/benzenoids, and fatty acid derivatives according to their biosynthetic pathways [4,5]. Terpenoids constitute the class with the largest secondary plant metabolites, and they are related to numerous biological activities [6].

Chrysanthemum is a famous traditional flower. The early study of its aroma focused on the functions of its essential oil, such as its antioxidant and antifungal functions [7,8]. However, the volatile compounds of chrysanthemum, such as *Chrysanthemum. indicum* var. *aromaticum* [9], cut chrysanthemum ‘Shen ma’ [10], and ‘gukhwa’ (*C. morifolium*), have recently been identified [11]. Although their scents are different, the main VOCs are terpenoids and their oxygen derivatives, especially limonene, pinene, and camphor [12]. The entire plant of *O. taihangensis* releases a pleasant and strong fragrance, which is completely distinguished from that of other species of chrysanthemum. The compound or series of compounds responsible for the production of the special leaf fragrance of *O. taihangensis* have not yet been investigated.

VOCs are commonly released from specialized tissues, such as laticifers, the secretion of cells, the resin canal, and trichomes [13]. Plant trichomes, as the special structures found on the aerial surface of plants, can be divided into glandular trichomes (GTs) with a secretory function and non-glandular trichomes (NGTs) without this function. Glandular trichomes are found in 30% of vascular plants, especially dicotyledons [14]. Different volatile compounds are accumulated in different types of GTs [15]. Monoterpenoids from peppermint are mainly enriched in peltate-type trichomes, which have a large subcuticular cavity [16]. *Ocimum* also synthesizes and accumulates mono- and sesquiterpenes in capitate and peltate trichomes [17]. In *Solanum*, specific metabolites are produced by different subtypes of capitate trichomes. In particular, the type VI glandular trichomes of *S. lycopersicum* are considered to be the main synthetic sites of terpenoids and flavonoids [18]. Calyx stalk trichomes are rich in cannabinoids, and monoterpenes are dominant in *Cannabis sativa* L. (cannabis) [19]. The number of trichomes also play a major role in the biosynthesis of volatiles [20]. In general, the content of specific metabolites increases as the density of GTs increases. In tomato, the greater density of type VI GTs can result in an increased content of terpenoids, which was detected in trichome-derived exudates [21]. Additionally, tobacco leaves with a higher density of GTs on the surface emitted a stronger aroma [22]. Due to the involvement of GTs in the biosynthesis of artemisinin, it is important to regulate GT initiation to improve the artemisinin contents in *Artemisia annua* [23].

The presence of NGTs and GTs is a common characteristic of Asteraceae plants [24,25], and the special scent of chrysanthemum leaves is produced by the GTs on the surface [26]. However, the relationship between the density of GTs and the released amounts of VOCs is poorly understood. As *O. taihangensis* is a wild species of the Asteraceae family with a unique fragrance, many researchers have focused on the pharmacological values of its methanol extracts, such as the anti-inflammatory and antiviral activities of *O. taihangensis*, rather than on the components and synthetic sites of the special aroma. Therefore, we aimed to identify the main volatile compounds of *O. taihangensis* leaves and to determine the role of GTs in aroma production by studying the density of GTs in relation to the content of aroma compounds. Dissecting the function of GTs in metabolites may provide a direction for aromatic breeding through the regulation of the capitate trichome initiation of *O. taihangensis* and *Chrysanthemum.*

## 2. Results

### 2.1. Identification of the Scent Components in Five Species

**Figure 1:**
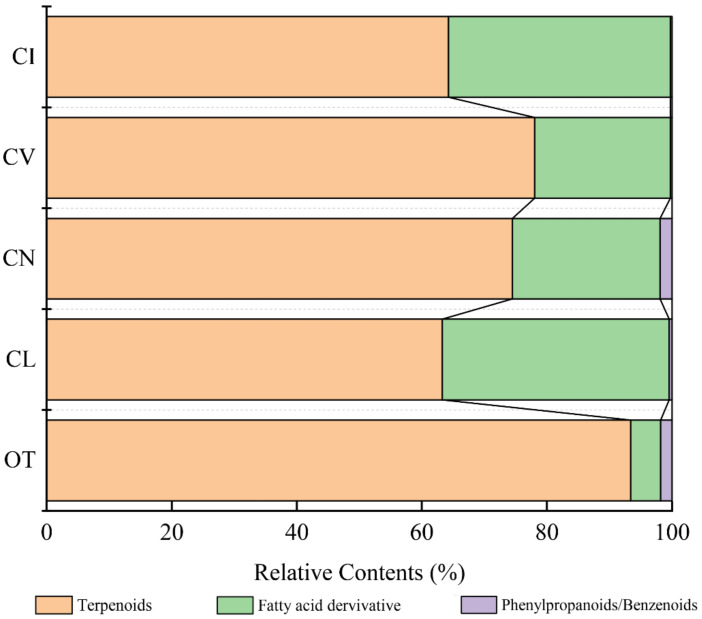
Comparison of the relative contents of the three major classes of scent compounds in the five species. Note: The names of the five materials in all figures and tables were replaced by abbreviations: OT—*Opisthopappus taihangensis*, CL—*Chrysanthemum lavandulifolium*, CN—*Chrysanthemum nankingense*, CV—*Chrysanthemum vestitum*, CI—*Chrysanthemum indicum*.

A total of 70 scent compounds, including terpenoids, phenylpropanoids/benzenoids, and fatty acid derivatives, were identified in five wild species by HS-SPME-GC/MS (Table S1). *Opisthopappus. taihangensis* had the most components (31)*,* whereas *C. indicum* had the least, with 17 compounds. In *C. vestitum, C. lavandulifolium*, and *C. nankingense,* 27, 19, and 20 components were detected, respectively. Among these compounds, terpenoids accounted for the largest proportion in every species, ranging from 63.28% in *C. lavandulifolium* to 93.41% in *O. taihangensis*. Fatty acid derivatives were emitted from the leaves of *C. lavandulifolium* and *C. indicum*, accounting for 36.27% and 35.44%, respectively, which were significantly higher than those from the other three species. Phenylpropanoids/benzenoids were found to have low emission amounts, accounting for only 0.27% (*C. vestitum*) to 1.92% (*C. nankingense*) (Figure 1).

**Figure 2:**
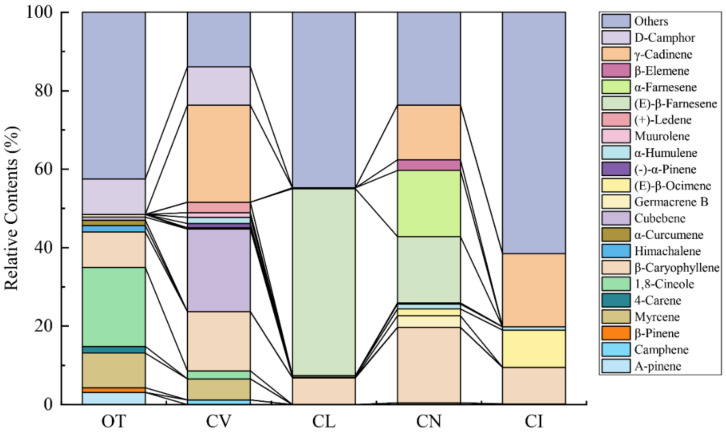
Major terpenoids (relative content >1%) emitted from the five wild species.

The principal components of the leaf volatile compounds differ obviously between the five species (Figure 2, Table S1). 1,8-cineole, myrcene, (-)-*α*-pinene, and himachalene contribute to the fragrance of *O. taihangensis* and create the unique fragrance of the *O. taihangensis* leaves. The 1,8-cineole is the principle volatile compound of the *O. taihangensis* leaves, which was found in smaller quantities or not at all in the leaves of the other four species. D-camphor, *γ*-cadinene, cubebene, and myrcene play important roles in the aroma emitted from *C. vestitum*. *γ*-cadinene accounts for the highest content (24.71%), and is also the main volatile compound of *C. indicum*. (E)-*β*-farnesene and *β*-caryophyllene are the principal constitutes of *C*. *lavandulifolium* and *C. nankingense*, respectively.

**Figure 3:**
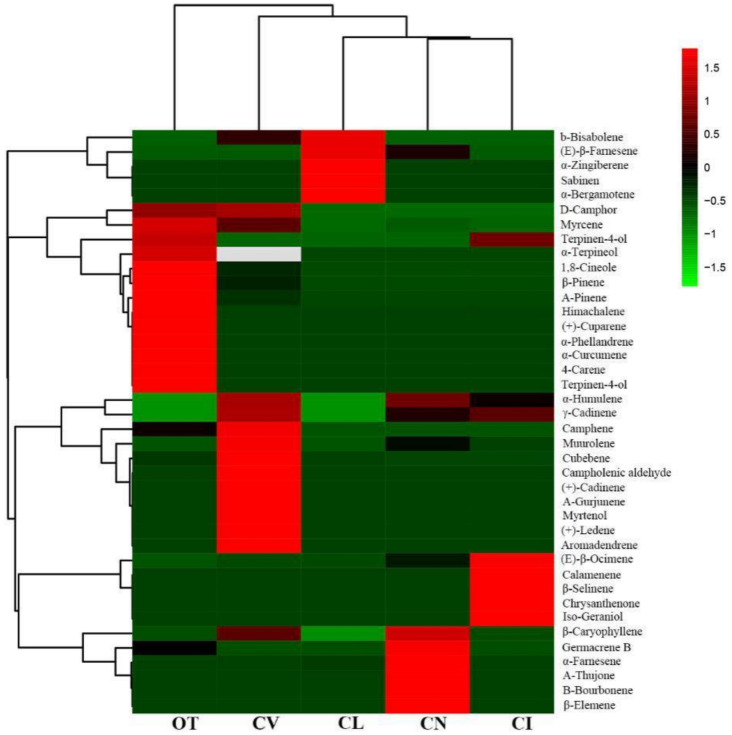
Clustering of terpenoids and the five wild species. The color scale of the heat map ranges from green (value, −1.5) to light red (value, 1.5). The emission values were normalized by log_10_ transformed.

Hierarchy cluster analysis was performed to analyze the relationships between the five species based on the main scent components. As shown in Figure 3, *O. taihangensis* was considered separately and showed considerable differences from the other four species in its aroma components. *Chrysanthemum lavandulifolium*, *C. nankingense*, and *C. indicum*, which are closely related species, were clustered together, and none of them have obvious odors. *C. vestitum* was also considered a separate one in the heat map clustering.

### 2.2. Principle Component Analysis (PCA) of the VOCs in Five Species

**Figure 4:**
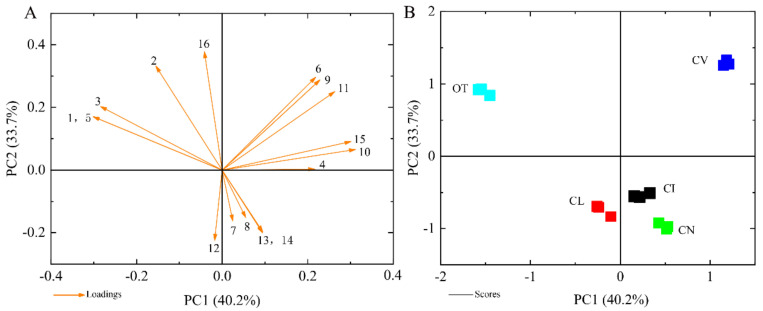
Principle component analysis (PCA) of the emission amount of the scent compound, considered separately for the five wild species. (**A**) The eigenvector load values of 16 scent components of five tested materials. (**B**) Principal component score chart for the five species. The numbers represent the major terpene compounds: 1, *β*-pinene; 2, myrcene; 3, 1,8-cineole; 4, *β*-caryophyllene, 5, himachalene; 6, cubebene; 7, germacrene B; 8, (E)- *β*-ocimene; 9, (-)-*α*-pinene; 10, *α*-humulene; 11, muurolene, 12, (E)-*β*-farnesene; 13, *α*-farnesene; 14, *β*-elemene; 15, *γ*-cadinene; 16, D-camphor. Data are presented from three independent biological replicates.

To show the differences in the main VOCs between the five species intuitively, PCA analysis was performed based on 16 major scent components. In Figure 4A, the first two principal components, PC1 and PC2, explained 40.2% and 33.7% of the variation, respectively. Volatiles with high positive scores in PC1 were *β*-caryophyllene, α-humulene, cubebene, (-)-*α*-pinene, muurolene and *γ*-cadinene, (Figure 4A), which might help to separate *C. vestitum* from the other taxa (Figure 4B). *β*-pinene, himachalene and 1,8-cineole showed negative values but high load scores in PC1, which could distinguish *O. taihangensis,* with an obvious odor, from the other four species (Figure 4B). The high loading values of myrcene, D-camphor, cubebene and (-)-*α*-pinene in PC2 made a distinction between *O. taihangensis*, *C. vestitum* and other three species. The principal component score plot did not overlap amongst the five wild species indicated that the composition and its relative content of scent compounds differed among different species.

### 2.3. Morphological Observation of GTs and NGTs

**Figure 5:**
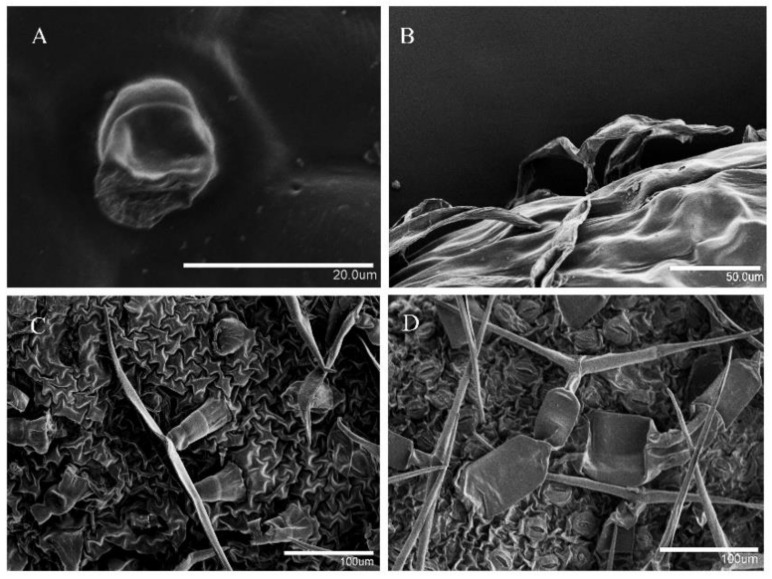
Micrograph observation of the leaf trichomes on the surface of leaves by SEM. (**A**) The capitate trichomes of *O. taihangensis*, and (**B**–**D**) the T-shaped non-glandular trichomes of *O. taihangensis*, *C. vestitum,* and *C. indicum*, respectively.

Scanning electron microscopy (SEM) was used to observe the types of trichomes on the surface of the leaves. The micrographs showed that there are two types of trichomes in the leaves of the five species: capitate and T-shaped non-glandular trichomes (Figure 5). As shown in Figure 5A, the capitate trichomes of *O. taihangensis* contain a basal cell, a shorter single-celled stalk, and apical secretory cells, which are usually comprised of two secretory cells arranged in the epidermis. Unlike the capitate trichome, the non-glandular trichome consists of two arm cells extending to a T shape, and this is why they are called T-shaped non-glandular trichomes. The morphologies of T-shaped trichomes differ in different species. On the surface of the leaves of *O. taihangensis*, the stalk cells are shorter and have twisted arm cells (Figure 5B). The arm cells of *C. vestitum* are straighter than those of the others (Figure 5C). The T-shaped part is composed of two to three stalk cells, and its arms are stretched at different angles and are more elongated than the arm cells of *C. vestitum* (Figure 5D). Due to the small amount of glandular and non-glandular trichomes on *C*. *lavandulifolium* and *C. nankingense* leaves, a micrograph observation of their trichomes is not shown in Figure 5.

### 2.4. Histochemistry of Capitate Trichomes

**Figure 6:**
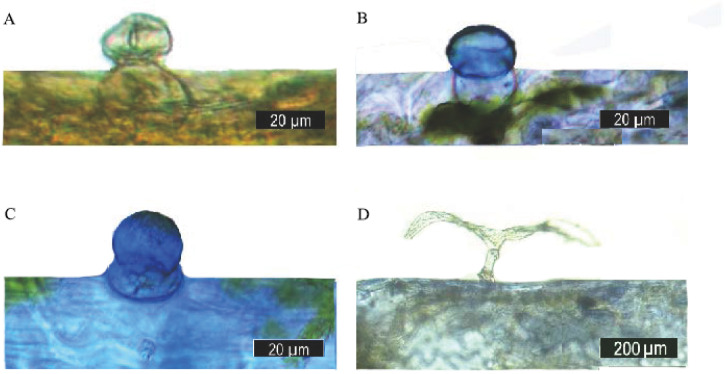
Histochemistry of the leaf glandular trichomes of *O. taihangensis*. (**A**) The capitate trichomes, before being stained. (**B**,**C**) positive Nadi reaction (blue-purple) in the head cell of a capitate trichome. (**D**) T-shaped non-glandular trichome without a reaction after staining.

To identify whether terpenoids accumulated in the capitate trichomes, the Nadi reaction was used to detect the secretory activities of the capitate trichomes and T-shaped non-glandular trichomes. In this study, sections of *O. taihangensis* leaves stained with Nadi showed that the secretory head of the capitate trichomes was stained violet, indicating the presence of terpenoids (Figure 6A–C). However, the T-shaped non-glandular trichomes remained colorless (Figure 6D). Thus, the capitate trichomes secrete terpenoids.

### 2.5. The Density of Trichomes in the Five Wild Species

**Figure 7:**
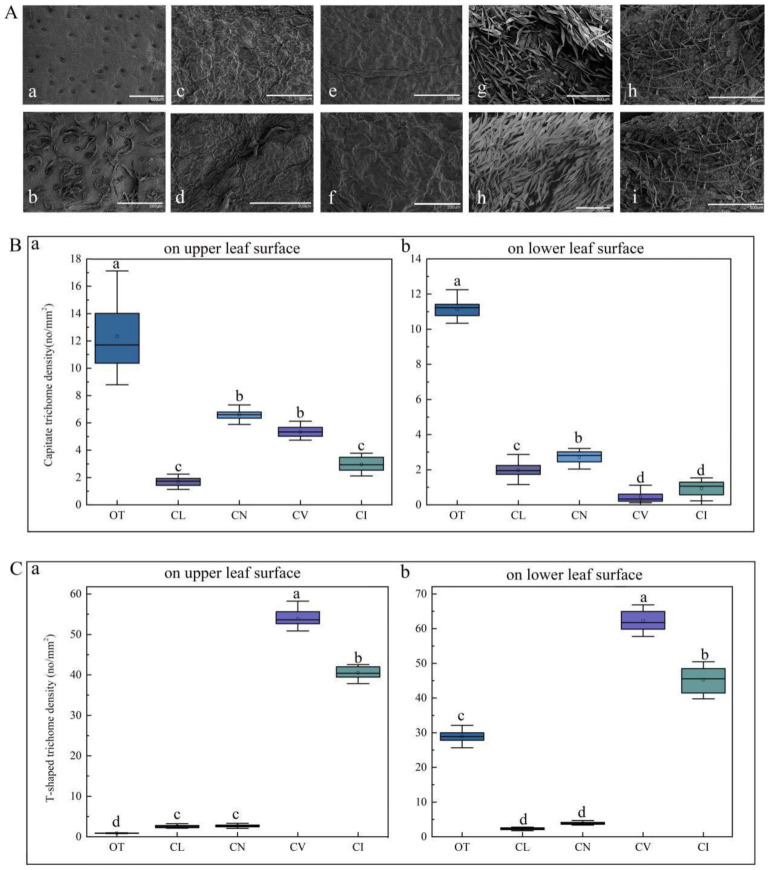
(**A**) The distribution of leaf trichomes on the five wild species by SEM observation: (a) and (b), the distribution of the trichomes of *O. taihangensis* on the upper surface and lower surface, (c) and (d), the distribution of the trichomes of *C. lavandulifolium* on the upper and lower surface; (e) and (f), the distribution of the trichomes of *C. nankingense* on the upper and lower surface; (h) and (g), the distribution of the trichomes of *C. vestitum* on the upper and lower surface; (i) and (j), the distribution of the trichomes of *C. indicum* on the upper and lower surface. The density of (**B**) the capitate and (**C**) T-shaped trichomes on the leaves of the five wild species. The different letters (a–d) in the same column of (**B**,**C**) represent the significance among the different species, according to Tukey’s test (*p* < 0.05).

The distribution and density of trichomes were found to be different in these species through SEM observation and a one-way analysis of variance (ANOVA) statistical test. Figure 7A shows that the density of the capitate trichomes of *O. taihangensis* was the greatest on both the upper and lower epidermis (Figure 7A(a,b)), which is confirmed in Figure 7B. As shown in Figure 7A(c,d)–C, the surface of the leaves of *C. lavandulifolium* had the fewest trichomes, including capitate and T-shaped trichomes. Similarly, only a few trichomes appeared on the surface of the leaves of *C. nankingense* (Figure 7A(e,f)). The capitate trichomes on the upper surface were rarely more numerous than those on the lower surface (Figure 7B). However, there were no significant differences between the number of capitate trichomes on the upper surface of *C. nankingense* and those on *C. vestitum*, which was higher than those on *C*. *indicum* (Figure 7B). The leaves of *C. vestitum* and *C. indicum* were almost covered by T-shaped trichomes (Figure 7A(g–j)), and there were far more T-shaped than capitate trichomes (Figure 7B,C). The density of T-shaped trichomes on the leaves of *C. vestitum* and *C. indicum* was obviously greater than on other species (Figure 7C). The difference in the density of the T-shaped trichomes in *C. nankingense* and *C. lavandulifolium* was not obvious (Figure 7C).

### 2.6. The Relationship between Volatile Content and Glandular Density

**Figure 8:**
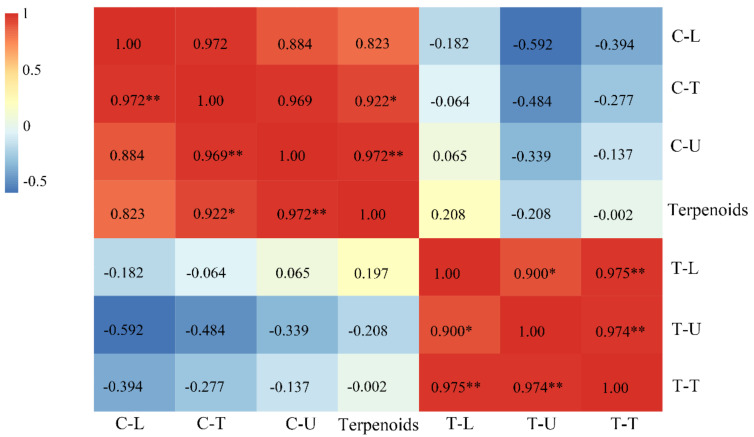
Correlation analysis of the content of terpenoids and the density of trichomes on the surface of leaves (T-shaped and capitate trichomes). **, *p* < 0.01; *, *p* < 0.5 level; C-U, C-L, and C-T, the density of capitate trichomes on the upper, lower and whole surface (total density of capitate trichomes) of leaves, respectively; T-U, T-L, and T-T; the density of T-shaped trichomes on the upper, lower and whole surface (total density of T-shaped trichomes)of the leaves, respectively. Terpenoids represent the content of terpenoids on the leaves.

**Figure 9:**
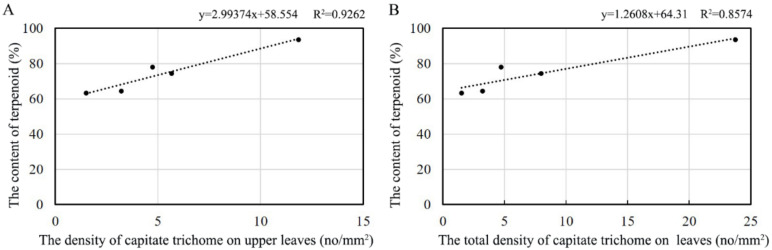
Regression analysis of the leaf terpenoid contents and the density of trichomes on (**A**) the upper leaf surface and (**B**) the whole leaf surface.

As reported, terpenoids are secreted by capitate trichomes [26]. To explain the relationship between the content of volatile compounds and the density of trichomes, a correlation analysis was conducted. The results showed that a significant correlation exists between the contents of terpenoids and the density of capitate trichomes on the whole leaf and the upper leaf surface of the leaves, with correlation coefficients of 0.972 and 0.922, respectively (Figure 8). However, we found no significant correlation with the density of the capitate trichomes on the lower leaf surface. The density of T-shaped trichomes is unrelated to the content of terpenoids, either on the upper or the lower leaf surface. The regression model of leaf terpenoid contents and the density of capitate trichomes showed a linear regression, and the correlation coefficients were 0.9262 and 0.8574, respectively, reaching the most significant level (Figure 9).

## 3. Discussion

Volatile compounds, such as polyphenols, polysaccharides, flavonoids, and alkaloids, play important roles in communication and defense in plants [25]. Our research showed that terpenoids are the class of compounds that are mainly emitted by the leaves of the five wild species, and the amount of terpenoid emissions from the *O. taihangensis* leaves is the greatest. As one of the largest classes of secondary metabolites, not only do terpenoids produce various aromas, but they are also related to numerous biological activities, such as plant defense and pollination, and they could be widely used in essential oils and medicine [27].

Many studies show that the VOCs in chrysanthemum flowers include β-pinene, eucalyptol, camphor, borneol, bornyl acetate, etc. [28,29]. However, there are some differences in the aroma components between chrysanthemum and its wild relative. In our study, the composition of the volatiles from the leaves of five wild species was analyzed. The aroma of *O. taihangensis* was significantly unique compared to the others. Among the 31 volatile compounds detected in *O. taihangensis*, 1,8-cineole was the main substance in *O. taihangensis*, and it was either found in small amounts or was not detected in the leaves of the other wild species. The substance 1,8-cineole, with a grass and mint smell and a camphor flavor [30], is one of the main ingredients of lavender oil. It contributes to the floral fragrance and flavor of *Artemisia frigida*, *C. coronarium,* and kiwifruit [31,32]. The sensory profile of 1,8-cineole is described as having significant “eucalyptus, mint, pine, and flowery” notes [33]. Thus, 1,8-cineole is considered an important contributor to the unique aroma of *O. taihangensis*. Terpinyl acetate, with a bergamot flavor; *γ*-terpinene, with a citrus aroma, cinene; and bornyl acetate (pine camphor notes) also play important roles in forming the unique scent of *O. taihangensis* leaves. All these terpenoids cause the characteristic flavor of *O. taihangensis* (cool and sweet) and its medicinal properties. The 1,8-cineole was also detected in the leaves of *C. vestitum,* which has a smell that is slightly cool. *C*. *lavandulifolium* and *C. nankingense* belong to the *C. indicum* group at the diploid level, but *C. vestitum* does not [34]. Hierarchy cluster analysis also showed that *C*. *lavandulifolium*, *C. nankingense*, and *C. indicum* are clustered together (Figure 3), and the two species also share the same main compound, (E)-*β*-farnesene, which has a green fragrance. This further demonstrates that they are closely related species, but *C. vestitum* is separate.

Investigations show that GTs and NGTs are widely distributed in plant species, such as *Medicago sativa* [35], *Humulus lupulus* [36], *Cannabis sativa* [37], *Lycopersicum esculentum* [38], *Nicotiana tabacum* [39], etc. Non-glandular trichomes form the first barrier preventing the invasion of herbivores and pathogens [40]. Glandular trichomes are variously shaped and have different functions in terms of their biological activities. Usually, a GT is composed of a glandular base, aerophore, and glandular head that determine its function of producing secretions [15]. Additionally, the obvious difference between GTs and NGTs is the secretion function. In *A. annua*, AaGSTs (secretory GTs), with a 10-celled biseriate translucent structure of four subapical cells and two apical cells, can secrete artemisinin [41]. However, among the eight different types of *Solanaceae* species, only four types (types II, III, V, and VIII) are secretory GTs [38]. In Asteraceae plants, *Helianthus annuus* [42], *Pyrethrum cinerariaefolium* [36], and *A. annua* [43] also have GTs with a secretion function. The GTs of *Tussilago farfara* have an oblong head and a long stalk cell and can produce phenols and terpenoids [44]. We observed the trichome types and distribution in the leaves of five wild species of chrysanthemum, and the results showed that there are two morphologically distinct trichome types: T-shaped trichomes (NGTs) and capitate trichomes (GTs) with a secretory function. Histochemical staining also revealed that the head cell of capitate trichomes contains terpenoids, which is consistent with the function of capitate trichomes [45].

The physical characteristics, such as the size and density, of GTs can affect the production or secretion of secondary metabolites [46]. In particular, the relationship between the formation and secretion of volatile oils and the density of GTs has been discussed in relation to many plants, including peppermint [47,48], lavender [49], and tomato [42]. Usually, plants with a larger GT density have a relatively stronger aroma. In this study, relative and regression analysis showed that the relative amounts of terpenoids have a close relationship with the density of the capitate trichomes on the upper and the whole leaf surfaces. As in this study, researchers have shown that the density of GTs is positively correlated with the production of compounds in *A. annua* [50,51]. Thus, we speculate that the rich aroma of *O. taihangensis* leaves might be due to the high density of the capitate trichomes on the surface of the leaves.

Aromatic oil extracts of *O. taihangensis* have broad development potential and application prospects. Thus, increasing the density of GTs to enhance the contents of natural metabolites is an effective strategy [23]. Some key genes involved in the biosynthesis of terpenoids show trichome-specific patterns in some plants, such as *ADS* [52], *CYP71AV1* [53], *STTPS5* [54], etc. Many transcription factors can promote the biosynthesis of terpenoids by positively regulating the key genes of the synthesis of terpenoids. In *A. annua*, many trichome-specific transcription factors, such as *AaGSW1* [55], *AaMIXTA1* [23], and *TAR1* [56], can regulate *CYP71AV1* expression to improve the content of artemisinin. As GTs are regarded as phytochemical factories, region insights into trichome-specific genes and transcription factors could help to elucidate the regulatory mechanism of the biosynthesis of terpenoids. The correlation between the density of capitate trichomes and the terpenoid contents in *O. taihangensis* leaves was evaluated. However, little is known about the molecular mechanism of the synthesis of terpenoids through the regulation of the capitate trichomes’ initiation in *O. taihangensis* and chrysanthemum. This still needs to be further investigated.

## 4. Materials and Methods

### 4.1. Plant Materials

*O. taihangensis, C. indicum, C. nankingense, C. lavandulifolium*, and *C. vestitum* were taken from the annual cutting of seedlings grown in the phytotron at the breeding farm of the National Flower Engineering Technology Research Center (Beijing, China), with a photoperiod of 16 h light/8 h dark at 25 °C and 65% RH (relative humidity). When the plants grew to a height of 10–15 cm, the third leaves in the plants of the 5 species were collected for the following experiments. The names of the five materials in the figures and tables were replaced by abbreviations: OT (*O. taihangensis*), CI (*C. indicum*), CN (*C. nankingense*), CL (*C. lavandulifolium*), and CV (*C. vestitum*).

### 4.2. Headspace Solid-Phase Micro-Extraction (HS-SPME) Sampling

The volatile compounds were detected mainly by using HS-SPME. Before the first use, the 50/30 μm of DVB-CAR-PDMS-2 cm SPME fiber (Supelco, Bellefonte, PA, USA) was adjusted to 2 h at 250 °C in the GC injection port. Approximately 0.1 g fresh weight of leaves of each species was placed in a head-space bottle (volume 20 mL) and allowed to stand for 10 min at an ambient room temperature and the empty capped vial was used as the blank control. An SPME device was inserted into the upper side of the sealed vial by manually penetrating the silicone septum. The volatiles were absorbed at room temperature (25 ± 5 °C) for 60 min. After extraction, the fibers were absorbed for 5 min at 250 °C in the injection plot and then analyzed by GC-MS analysis (GC-2000/Mars-6100 GC/MS combined instrument; condenser technology, Hangzhou Co., Ltd. Manufacturing, China). The experiment was conducted in triplicate.

### 4.3. GC and MS Conditions

Volatile compounds were analyzed using a GC-MS system, equipped with a DB-5MS quartz capillary column (30 m × 0.25 mm × 0.25 µm, Shimadzu). The column oven temperature program was as follows: 40 °C for 2 min, increasing to 200 °C at a rate of 5 °C/min and holding for 6 min. Helium (99.999%) was used as the carrier gas at a flowrate of 1 mL/min. The ion source and quadrupole temperatures were 230 and 150 °C, respectively. The ionizing energy was 70 eV, and the mass range scanned was 40–500 amu in the full-scan acquisition mode.

### 4.4. Scanning Electron Microscopy (SEM) Observation

Small pieces (3 × 3 mm) of fresh leaves were fixed in an FAA fixative (formaldehyde: glacial acetic acid: 70% alcohol, 5:5:90, by volume). To completely immerse the tissue samples in the fixation liquid, they were extracted with a vacuum pump at 1 kpa for 30 min. Before critical point drying (CPD), the samples were washed 3 times (each time for 10 min) with a phosphate buffer (PBS pH = 6.8) and dehydrated through an ascending ethanol series (30%, 50%, 70%, 90%, and 100%, each time for 15 min) and in 100% acetone. This was followed by stepwise rinsing with 100% isoamyl acetate for 15 min. Then, the samples were treated by vacuum spray plating. An S-3400N scanning electron microscope (Hitachi, Japan) was used to observe the trichomes, and fluorescence microscopy (Leica M165FC, Germany) was used to determine the density of both the GTs and NGTs on the surface of the leaves. The determination was repeated 20 times: 5 individuals, with 4 visual field repetitions per leaf.

### 4.5. Histochemical Characterization

Histochemical analyses were performed on a hand-section of fresh leaves. Terpenoids that existed in glandular trichomes were investigated using the Nadi reaction. The leaves were placed for 30 min in a freshly prepared Nadi buffer (10% 1-naphthol, 40% ethanol, and 1% dimethyl para-phenylenediamine dihydrochloride in a 0.05 M phosphate buffer) [57]. Then, the stained trichomes were observed with the Prog Res C5 (Leica, Germany), and images were captured.

### 4.6. Statistical Analysis

The total ion current was obtained by integrating the peak area of every scent compound. The volatile compounds in the leaves of the five wild species were identified by comparison of their mass spectra with the compounds present in the NIST11 library (the National Institute of Standards and Technology 2011, Shimadzu, Japan) using GC/MS Postrun Analysis software. Area normalization was used to determine the relative content of volatile compounds and normalization from Equation (1) [58]. Based on the data, which were log_10_ transformed, clustering of the relative content of terpenoid compounds and the five wild species was conducted using the R software, pheatmap (version 3.5.0). Principal component analysis (PCA) was performed using Origin software (MicroCal, USA, 2018) to calculate the eigenvector load values and identify the major floral scent components (with a relative content greater than 1%). The Nano software program was used to count the number of GTs and NGTs present on the surface of the leaves and calculate the density from the field of view through selected (the number of glandulars/field of view) images derived from fluorescence microscopy (Figure S1). All statistical analyses were performed using IBM SPSS software (IBM SPSS Satistics Inc, version 20; Chicago, IL, USA). One-way analysis of variance (ANOVA) was used to test the significance at a significance level of *p* ≤ 0.05. Regression analysis was performed to analyze the correlation between the volatile amounts and the density of the glandular trichomes on the surface of the leaves.

(1) $$Relative\;content\;\left(\%\right)=\frac{area\;under\;peak}{total\;peak\;area}\times 100$$

## Supplementary Materials

The following are available online at https://www.mdpi.com/2223-7747/9/7/855/s1, Table S1: The volatile organic compounds of five species.
