# Supplementary material for: Identification of the Volatile Compounds and Observation of the Glandular Trichomes in Opisthopappus taihangensis and Four Species of Chrysanthemum
Source: Plants (Basel). 2020 Jul 6;9(7):855. doi: 10.3390/plants9070855 (PMC7412243; doi:10.3390/plants9070855)
Supplement: Supplementary file 1 [file plants-09-00855-s001.pdf]

plants

MDPI

SUPPLEMENTARY INFO

Identification of Volatile Compounds and Observation of Glandular Trichomes in *Opisthopappus taihangensis* and Four Species of *Chrysanthemum*

Yanhong Guo[1], Tengxun Zhang[1], Jian Zhong[1], Tingting Ba[1], Ting Xu[1], Qixiang Zhang[1,2] and Ming Sun[1*]

1. Beijing Key Laboratory of Ornamental Plants Germplasm Innovation & Molecular Breeding; National Engineering Research Center for Floriculture; Beijing Laboratory of Urban and Rural Ecological Environment; Key Laboratory of Genetics and Breeding in Forest Trees and Ornamental Plants of Ministry of Education; School of Landscape Architecture, Beijing Forestry University, Beijing, China
2. Beijing Advanced Innovation Center for Tree Breeding by Molecular Design, Beijing Forestry University, Beijing, 100083, China.

Table 1. Main volatile compounds of five wild species.

Compounds	CAS	Relative content (%)				
		OT	CV	CL	CN	CI
Leaf alcohol	928-96-1	1.01±0.03	15.40±1.25	13.77±1.17	12.66±0.93	11.35±0.94
(+)-Cuparene	16982-00-6	0.26±0.03	-	-	-	-
α-Phellandrene	99-83-2	0.2±0.02	-	-	-	-
(-)-α-Pinene	80-56-8	3.1±0.25	0.19±0.08	-	-	-
Camphene	79-92-5	0.36±0.03	1.17±0.15	-	-	-
β-Phellandrene	555-10-2	4.23±0.3	0.66±0.06	-	-	-
β-Pinene	18172-67-3	1.22±0.07	0.16±0.07	-	-	-
Methylheptenone	110-93-0	0.27±0.02	-	-	-	-
Myrcene	123-35-3	8.83±0.12	5.4±0.9	-	0.43±0.07	0.18±0.04
Leaf acetate	3681-71-8	1.3±0.17	8.6±0.64	4.51±0.74	5.67±0.84	-
4-Carene	29050-33-7	1.67±0.22	-	-	-	-
o-CyMene	527-84-4	1.56±0.15	-	-	-	-
1,8-Cineole	470-82-6	20.15±0.15	2.00±1.00	-	-	-
Phenylacetaldehyde	122-78-1	0.24±0.07	-	-	-	-
γ-Terpinene	99-85-4	0.45±0.079	-	-	-	0.32±0.51
Trans-sabinene hydrate	17699-16-0	1.3±0.27	-	-	-	0.45±0.02
Terpinolene	586-62-9	0.38±0.15	-	-	-	0.33±0.23
D-Camphor	464-49-3	9.02±0.78	9.8±0.95	-	-	-
Terpinen-4-ol	562-74-3	0.32±0.1	-	-	-	-

Methyl salicylate	119-36-8	0.51±0.18	-	0.68±0.11	0.06±0.02	0.07±0.03
α-Terpineol	98-55-5	0.87±0.11	-	-	-	-
Cis-3-Hexen-1-yl Valerate	35852-46-1	0.20±0.08	-	-		-
3-methyl-6-(1-methylethyl)-2-Cyclohexen-1-one	89-81-6	1.74±0.17	-	-	-	-
Terpinyl acetate	80-26-2	7.30±0.53	-	-	-	-
β-Caryophyllene	87-44-5	9.06±0.19	15.15±0.54	6.86±0.33	19.27±0.66	9.34±0.33
Himachalene	1461-03-6	1.62±0.17	-	-	-	-
α-Curcumene	644-30-4	1.34±0.12	-	-	-	-
Cubebene	13744-15-5	0.83±0.09	21.02±1.42	0.18±0.08	-	-
7-Heptadecene	56554-78-0	0.46±0.11	-	-	-	-
Germacrene B	15423-57-1	0.68±0.18	-	-	2.90±0.46	-
1-Chlorooctadecane	3386-33-2	0.28±0.16	-	-	-	-
1-Octen-3-ol	3391-86-4	-	0.36±0.11	-	-	-
3-Octanone	106-68-3	-	0.35±0.09	-	-	-
(E)-β-Ocimene	3779-61-1	-	0.34±0.09	0.36±0.09	1.76±0.12	9.47±0.2
Campholenic aldehyde	4501-58-0	-	0.17±0.07	-	-	-
Myrtenol	515-00-4	-	0.14±0.06	-	-	-
Cis-3-Hexen-1-yl Valerate	35852-46-1	-	0.25±0.08	-	-	-
a-Gurjunene	489-40-7	-	0.18±0.07	-	-	-
α-Humulene	6753-98-6	-	1.55±0.23	-	-	0.79±0.11
Alloaro Madendrene	25246-27-9	-	0.59±0.16	-	-	-
Gamma muurolene	30021-74-0	-	1.21±0.14	-	0.26±0.08	0.07±0.1
(+)-Ledene	21747-46-6	-	2.67±0.21	-	-	-
Cyclohexane	3242-08-8	-	5.26±0.15	-	-	-
(+)-Cadinene	483-76-1	-	1.64±0.11	-	-	-
b-Bisabolene	495-61-4	-	0.19±0.06	0.43±0.12	-	-
Hexyl acetate	142-92-7	-	0.45±0.11	0.34±0.08	0.26±0.08	-
(Z)-3-Hexenal	6789-80-6	-	-	2.95±0.23	-	-
Sabinen	3387-41-5	-	-	0.17±0.06	-	-
Phenethyl alcohol	60-12-8	-	-	0.14±0.07	0.07±0.01	-
Ethyl phenylacetate	101-97-3	-	-	0.66±0.11	-	-

Acetic acid linalool ester	115-95-7	-	-	0.86±0.09	-	-
(E)-β-Farnesene	28973-97--9	-	-	47.61±0.20	16.89±0.21	-
α-Cedrene	469-61-4	-	-	0.11±0.04	-	-
Nerolidol	7212-44-4	-	-	0.10±0.05	-	0.23±0.11
α-Bergamotene	17699-05-7	-	-	0.86±0.10	-	-
α-Zingiberene	495-60--3	-	-	0.24±0.09	-	-
α-Farnesene	502-61-4	-	-	0.25±0.08	16.89±0.1	-
a-Thujone	546-80-5	-	-	-	0.07±0.03	-
Cis-3-hexenyl valerate	35852-46-1	-	-	-	0.82±0.08	-
B-Bourbonene	5208-59-3	-	-	-	0.05±0.01	-
β-Elemene	515-13-9	-	-	-	2.73±0.25	-
γ-Cadinene	39029-41-9	-	24.71±0.24	-	13.97±0.25	18.61±0.28
α-Humulene	6753-98-6	-	-	-	1.28±0.05	-
α-Patchoulene	560-32-7	-	-	-	0.10±0.03	-
Chrysanthenone	473-06-3	-	-	-	-	0.27±0.07
Iso-Geraniol	5944-20-7	-	-	-	-	0.09±0.02
(-)-Isocaryophyllenel	118-65-0	-	-	-	-	0.35±0.04
β-selinene	17066-67-0	-	-	-	-	0.83±0.12
Calamenene	483-77-2	-	-	-	-	0.12±0.04

Note: '-' is not detected

OT—*Opisthopappus taihangensis*, CL—*Chrysanthemum lavandulifolium*, CN—*Chrysanthemum nankingense*, CV—*Chrysanthemum vestitum*, CI—*Chrysanthemum indicum*.

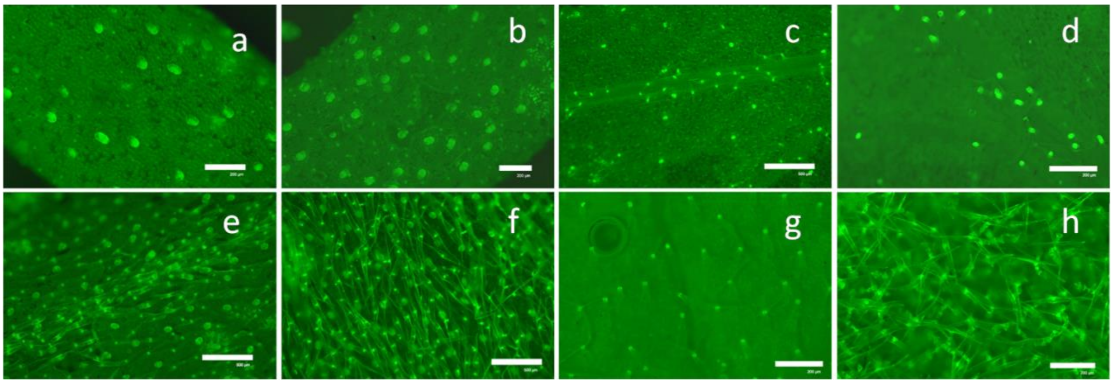

Figure 1. Fluorescence distribution of leaf trichomes of five wild species. a,b The distribution of trichomes of *O. taihangensis* on upper and lower surface. c,d The distribution of trichomes of *C. lavandulifolium* and *C.nankingense* on upper surface on upper and lower surface. e,f The distribution of trichomes of *C. vestitum* on upper and lower surface. g,h The distribution of trichomes of *C. indicum* on upper and lower surface.
